# Maternal screening coverage and determinants during facility-based antenatal care in Nigeria: analysis of the 2018 Demographic and Health Survey

**DOI:** 10.1186/s12978-026-02269-1

**Published:** 2026-01-28

**Authors:** Yusuf Adelabu, Tersur T. Saalu, Bosede B. Afolabi, Aduragbemi Banke-Thomas, Lenka Beňová

**Affiliations:** 1 Department of Infectious Disease Epidemiology and International Health, Faculty of Epidemiology and Population Health, London School of Hygiene and Tropical Medicine, London, UK; 2 Department of Medicine, Faculty of Clinical Sciences, College of Medicine, University of Lagos, Lagos, Nigeria; 3 Department of Obstetrics and Gynaecology, Lagos University Teaching Hospital, Idi-Araba, Lagos, Nigeria; 4 Department of Obstetrics and Gynaecology, Faculty of Clinical Sciences, College of Medicine, University of Lagos, Lagos, Nigeria; 5 Centre for Clinical Trials, Research and Implementation Science, University of Lagos, Lagos, Nigeria; 6 Maternal, Adolescent, Reproductive, and Child Health (MARCH) Centre, London School of Hygiene and Tropical Medicine, London, UK; 7 Department of Public Health, Institute of Tropical Medicine, Antwerp, Belgium

**Keywords:** Antenatal care, Coverage, Determinants, Health facility, Maternal health, Quality of care, Screening, Sector, Socio-economic determinants, Wealth inequality

## Abstract

**Background:**

Antenatal care (ANC) provides an opportunity for early identification and treatment of pregnancy-related conditions to prevent adverse maternal and foetal outcomes. The influence of socioeconomic and health system factors on maternal screening during ANC remains unclear. We evaluated the coverage and determinants of facility-based ANC use and components related to maternal screening for common pregnancy-related conditions in Nigeria.

**Methods:**

Using data from the 2018 Nigeria Demographic and Health Survey, we estimated the percentage of women aged 15–49 years who reported facility-based ANC for their most recent live birth in the 5 years preceding the survey. Among them, we estimated the percentage who received care components related to maternal screening (blood pressure measured, and urine and blood samples taken) at least once. We used logistic regression to examine the association between household wealth and 1) facility-based ANC utilisation and 2) screening components of care received. Facility type (private or public sector) was considered as an effect modifier.

**Results:**

Among the sample of 21,792 women, 72.8% reported facility-based ANC. Most ANC users reported having had their blood pressure measured (95.2%), and their blood (89.3%) and urine samples taken (88.0%). All three screening components were received by 83.4% of facility-based ANC users. Compared to women from the poorest quintile, the richest had significantly higher adjusted odds of having facility-based ANC (aOR 4.25, 95% CI: 3.23 – 5.59) and, among ANC users, of receiving all three screening components (aOR = 3.43, 95% CI: 2.52 – 4.68). The adjusted odds of receiving all three components were 49% lower among women who used private ANC compared to those who used only public providers (aOR = 0.51, 95% CI: 0.43 – 0.61). There was no evidence of interaction by facility type.

**Conclusion:**

Wealth inequality is associated with disparities in the utilisation of facility-based ANC and maternal screening. Socio-economically disadvantaged women, who are most in need of maternal health services, face a ‘double penalty’ of deprived ANC use and maternal screening during ANC. Interventions focused on mitigating these disparities can help improve maternal outcomes. Concerted efforts are required to regulate the private facilities and strengthen the public facilities to provide high-quality maternal healthcare services.

**Supplementary Information:**

The online version contains supplementary material available at 10.1186/s12978-026-02269-1.

## Abstract (plain language summary)

Pregnant women receive care in health facilities to ensure the well-being of themselves and their babies. The factors that affect the use and quality of care received by pregnant women are not well understood. We estimated the percentage of pregnant women who received care in health facilities and had tests to detect common pregnancy conditions, including measuring blood pressure, and taking urine and blood samples. Then, we examined whether the woman’s household wealth influences her ability to receive pregnancy care in health facilities and be tested for common pregnancy conditions. We also examined whether the type of facility where the women received care affects the tests done.

About three-quarters of the women reported receiving care in health facilities during pregnancy. Nearly all these women had their blood pressure measured, and approximately 9 in 10 had their blood and urine samples taken at least once. Women from the richest households were four times more likely to receive care in health facilities during pregnancy and three times more likely to have all three tests done compared to women from the poorest households. Women who had care in private facilities were about 50% less likely to receive all three tests than those in public facilities.

Differences in wealth status influence the ability of women to receive care in health facilities during pregnancy and tests to detect common pregnancy conditions, thereby facing a ‘double penalty’. Efforts are needed to eliminate these differences and improve the quality of care received in public and private facilities.

**Supplementary Information:**

The online version contains supplementary material available at 10.1186/s12978-026-02269-1.

## Background

Maternal health is a major global health priority, and its impact on population health extends beyond pregnancy, childbirth, and the postpartum period to the entire life course [1, 2]. The Sustainable Development Goal (SDG) target to reduce the global maternal mortality ratio (MMR) below 70 maternal deaths per 100,000 live births by 2030 is far from being achieved, despite a 40% reduction from 328 to 197 per 100,000 live births between 2000 and 2023 [3]. Sub-Saharan Africa accounted for 70% of the estimated 260,000 global maternal deaths in 2023, with the highest proportion from Nigeria (30%), where about 75,000 maternal deaths occurred [3]. Haemorrhage and hypertensive disorders are the leading direct causes of maternal mortality, with significant contributions from indirect causes, such as pre-existing hypertensive disorders and diabetes complicating pregnancy [4]. Most causes of maternal deaths are largely preventable and can be detected and treated effectively with available interventions [5].

Antenatal care (ANC) is an essential healthcare service provided by skilled healthcare professionals during pregnancy, aimed at ensuring the well-being of both the mother and her baby [6]. It offers an opportunity to prevent and detect pre-existing or pregnancy-related conditions through health education, health promotion, and risk identification. The World Health Organization (WHO) introduced the Focused ANC Model in 2002, recommending at least four ANC visits for every pregnant woman without any complications. This recommendation was revised in the 2016 WHO ANC Model to a minimum of eight ANC contacts based on evidence showing that the increased number of contacts was associated with greater maternal satisfaction and fewer perinatal deaths [7, 8]. The ANC contacts with skilled healthcare professionals occur in health facilities, such as clinics, health centres, or hospitals, and thus referred to as facility-based ANC. This excludes contacts with healthcare professionals outside a formal health facility, such as antenatal home visits, which aim to improve facility-based ANC use through mobilisation in areas with low maternal healthcare access [6].

Maternal screening and other risk assessments are performed during ANC visits in health facilities [5, 6]. The maternal screening components of ANC allow for early identification and management of pregnancy-related conditions, including anaemia, pre-eclampsia, gestational diabetes, and infections [6]. The WHO recommends that: blood pressure is measured, and urine is checked for protein to screen for pre-eclampsia at every ANC visit; urine is cultured for asymptomatic bacteriuria at the first visit; and blood is drawn for laboratory tests to screen for anaemia, gestational diabetes, haemoglobinopathies, and infections such as HIV, hepatitis, syphilis, and rubella, mostly during the first ANC visit but often conducted at specific intervals in pregnancy [6].

Despite the vital role of ANC in promoting maternal and foetal health, substantial inequities exist in access and utilisation, leading to adverse outcomes [2, 9]. Several social determinants of maternal health, including education, income, socioeconomic class, ethnicity, and sociocultural factors, have been identified [10]. These social determinants influence accessibility and utilisation of maternal health services, as well as the content and quality of care received, contributing to health disparities [9]. Wealth inequality and other socioeconomic disadvantages have been associated with increased maternal morbidity and mortality [10]. The health system plays a critical role in mitigating the negative effects of these socio-economic and socio-cultural factors on maternal health [10].

Nigeria is one of the eight sub-Saharan African countries in the “very high” maternal mortality category, with an estimated MMR of 993 maternal deaths per 100,000 live births in 2023 [3]. Despite a rising trend in ANC coverage from 58 to 67% in the last two decades, this has not translated into a significant reduction in maternal mortality [11]. This raises a question about the content and quality of care received during ANC visits in Nigerian health facilities. Studies from Nigeria and other sub-Saharan African countries have identified various socioeconomic factors that influence the utilisation of ANC and components of care [12–17]. However, little is known about maternal screening during ANC in Nigeria, and the factors influencing maternal screening for common pregnancy-related conditions remain unclear.

To achieve a reduction of the unacceptably high and preventable maternal deaths in Nigeria towards the SDG target, there is a need to ensure effective implementation of maternal screening components in ANC. This requires an understanding of the socioeconomic factors that influence the utilisation of screening services during ANC, which will be useful in developing strategies for effective implementation. This study aimed to estimate the coverage of facility-based ANC and components related to maternal screening for common pregnancy conditions, including blood pressure measurement, and the collection of blood and urine samples. Secondly, we examined the influence of household wealth on facility-based ANC utilisation and the relationship between household wealth and facility type on receipt of ANC components related to maternal screening.

## Methods

### Data

We used data from the 2018 Nigeria Demographic and Health Survey (NDHS), the most recent nationally representative survey on maternal and reproductive health indicators for Nigeria. The NDHS provides national, regional, and state-level information on population and health. It was first conducted in 1990 by the National Population Commission, with support from ICF, and has been repeated every five years since 2003. The sampling design involves administrative units, including local government areas, states, and enumeration areas derived from the most recent census. In a stratified two-stage cluster design, 1400 enumeration areas were first selected, followed by the selection of 30 households in each enumeration area. In total, 42,000 households were selected with 42,121 eligible women aged 15–49 years present either as permanent residents of the selected households or as visitors the night preceding the survey. Trained field interviewers administered a computer-assisted, structured questionnaire to the women face-to-face. Data for the 2018 NDHS were collected between August and December 2018 from 41,821 eligible women, corresponding to a 99% response rate [11].

### Study population

We included all women aged 15 to 49 who had a live birth in the five years preceding the survey to evaluate coverage and determinants of facility-based ANC utilisation. We assessed ANC use and components of ANC for the most recent live birth. Among women who had at least one ANC visit in a health facility during pregnancy for their most recent live birth in the recall period, we assessed the coverage and determinants of receiving the components related to maternal screening.

### Outcome variables

We assessed two main outcome (dependent) variables in a two-step analysis (Table 1). The first outcome was facility-based ANC use, defined as reporting at least one ANC visit in a health facility during pregnancy for the most recent live birth. The women were asked in the survey about the place of ANC, and the interviewers probed for all locations, including homes and health facilities, allowing multiple responses. We regarded the receipt of ANC in the following locations to establish facility-based ANC use: government hospitals, health centres, health posts, or other public sector facilities, as well as private hospitals, clinics, or other private medical sector facilities. Although the 2016 WHO ANC Model recommends at least eight ANC contacts, we defined adequate ANC in this analysis as four or more visits during pregnancy, reflecting the Focus ANC Model that was the recommendation for most of the survey recall period (2013–2018). Timely ANC refers to having the first ANC visit within four months of pregnancy. Both timely and adequate ANC visits were considered in the context of facility-based ANC use. Women who did not report the number of ANC visits and timing of their first ANC visit were reclassified as ANC non-users. Women who reported the timing of their first ANC visit but did not report the number of visits were included as ANC users, but without adequate ANC. The second outcome in this analysis was the receipt of all ANC components related to maternal screening for common pregnancy conditions, including: (1) blood pressure measured, (2) urine sample taken, and (3) blood sample taken, at least once during ANC for their most recent live birth, among facility-based ANC users.

**Table 1:** Outcome variables for antenatal care (ANC) use and maternal screening components in ANC

Outcome variable	NDHS question	Categories
All women with a live birth:		
Any ANC visit	Did you see anyone for antenatal care for this pregnancy?	Yes (1); No (0)
Location of ANC	Where did you receive antenatal care for this pregnancy? (Government hospital, health centre, health post, or other public sector facility, and private hospital/clinic or other private medical sector were regarded as health facilities)	Health facility (1) Other (0)
Facility-based ANC*	Combined variable (Any ANC + ANC in health facility)	Yes (1); No (0)
Women with any facility-based ANC visit:		
Timely ANC	How many months pregnant were you when you first received antenatal care for this pregnancy?	≤ 4 months (1) > 4 months (0)
Adequate ANC	How many times did you receive antenatal care during this period?	≥ 4 visits (1) < 4 visits (0)
Timely and adequate ANC	Combined variable (timely ANC + adequate ANC)	Yes (1); No (0)
Women with facility-based ANC:		
Components related to screening:	As part of your antenatal care, were any of the following done at least once:	
Blood pressure measured	Was your blood pressure measured?	Yes (1); No (0)
Urine sample taken	Did you give a urine sample?	Yes (1); No (0)
Blood sample taken	Did you give a blood sample?	Yes (1); No (0)
All three components*	Blood pressure measured, AND urine sample taken, AND blood sample taken	Yes (1); No (0)

^*^Main outcome variables

### Exposure variables

**Fig. 1:**
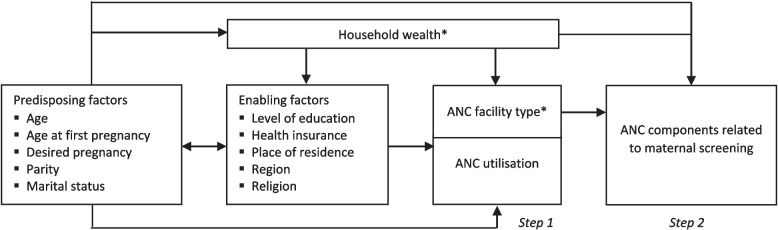
Conceptual model of potential factors influencing facility-based ANC utilisation and receipt of maternal screening components. *Main exposure variables

The exposure (independent) variables are potential factors influencing facility-based ANC utilisation and receipt of ANC components related to maternal screening, organised in a conceptual model (Fig. 1). This conceptual model was adapted from Anderson’s behavioural model of health service utilisation, which categorises the predictors into predisposing characteristics (inclination to use health services), enabling factors (ability to seek health services), and need factors (perceived need of health services) [18]. The model has previously been adopted to assess determinants of maternal health service utilisation in resource-limited settings [19, 20].

In a two-step analysis, we initially evaluated household wealth as the primary exposure for facility-based ANC use among all women who required ANC, then examined household wealth and facility type as the main exposures for receiving ANC components related to maternal screening among ANC users (Fig. 1). Household wealth was retained in the second step to compare its association with facility-based ANC utilisation and receipt of the ANC components. However, because it is the responsibility of the health system to perform the ANC components when women access ANC, we also examined the association between facility type and receiving ANC components related to maternal screening. Household wealth was calculated in the NDHS as scores derived from primary component analysis of household assets for the household population, and categorised as wealth index quintiles from poorest to richest [11]. For the facility type, we categorised health facilities according to the NDHS questionnaire by sector into public (government hospital, health centre, health post, and other public sector facilities) and private (hospital, clinic, and other private medical sector facilities). Women who had ANC in public and private or only private health facilities were regarded as having ANC in any private facility, while the other category comprised women who had ANC in only public facilities [21].

Furthermore, we evaluated the interaction between household wealth and facility type on the receipt of ANC components related to maternal screening. In the Nigerian health system, which has a low health insurance coverage, most health services are financed out-of-pocket [22]. Public health facilities in Nigeria typically offer subsidised or sometimes free health services through public or donor funds, thereby making out-of-pocket payments generally more affordable than in private health facilities [23]. We hypothesised that poorer women who had ANC in private facilities were less likely to receive all components related to maternal screening than wealthier women who had ANC in private facilities, compared to public facilities.

### Other independent variables

All other predisposing and enabling factors in the conceptual model were included as confounders in the analysis. The description and categories of the independent variables are shown in Table 2.

**Table 2:** Other independent variables for facility-based ANC utilisation and components related to maternal screening

Variable	Description	Categories
Age at most recent live birth	Grouped based on obstetric risk	15–19, 20–34, 35–49
Age at first pregnancy	Adolescent during their first pregnancy or not	< 20, ≥ 20
Parity	Number of children before the most recent live birth	0, 1, 2–4, 5 +
Desire for a baby	The woman’s desire for the most recent birth	Wanted then, wanted later, or wanted no more
Marital status	Marital status at time of interview	Currently in a union or not
Educational level	Highest educational level attended or completed	None, primary, secondary, or higher
Health insurance	Health insurance coverage for the woman	Covered or not
Place of residence	Current location of the woman’s household as defined in the DHS	Rural or urban
Region	Geopolitical zone of residence	North-Central, North-East, North-West, South-East, South-West, or South-South
Religious affiliation	The woman’s self-reported religion	Christianity, Islam, Traditional/others

### Data analysis

All statistical analyses were performed on the 2018 NDHS women dataset using Stata version 18.0 Standard Edition (StataCorp, College Station, Texas, USA). The *svyset* command accounted for clustering, stratification, and sampling weights applied in the complex survey design. Descriptive statistics, which showed the distribution of all independent variables for the study, were calculated as frequencies and percentages for all women with live births and facility-based ANC users. We estimated the percentage of women with live births who had facility-based ANC and the percentage of facility-based ANC users who received individual and combined ANC components related to maternal screening. Coverage data for facility-based ANC and maternal screening components were visualised across wealth quintiles using Stata codes from Equiplot, a tool designed to illustrate equity data [24].

Bivariate analyses were carried out using logistic regression to investigate the association between the outcomes of interest and the exposure variables. Then, multivariable logistic regression was used to examine the association between household wealth and facility-based ANC utilisation, adjusting for other independent variables as confounders. Next, multivariable logistic regression models were generated to determine the independent and interaction effects of household wealth and facility type on receipt of ANC components related to maternal screening, adjusting for other independent variables as confounders. We adjusted for timely and adequate use of facility-based ANC as a confounder to receiving ANC components related to maternal screening. Crude and adjusted odds ratios (OR), along with the corresponding 95% confidence intervals (CI), were calculated. To avoid overlap, the independent variables were checked for collinearity using the Variance Inflation Factor (VIF), with a threshold of less than 2. The fit of the multivariable regression and interaction models was checked using a design-based Wald statistic with the *test* command.

### Ethical considerations

The ethical clearance required before conducting the survey was obtained from the National Health Research Ethics Committee in Nigeria and the ICF Institutional Review Board. All survey participants gave informed consent. Measure DHS granted access to the dataset, and the Research Ethics Committee of the London School of Hygiene and Tropical Medicine approved this secondary analysis of the anonymised data (LSHTM Ethics Ref: 31568).

## Results

**Fig. 2:**
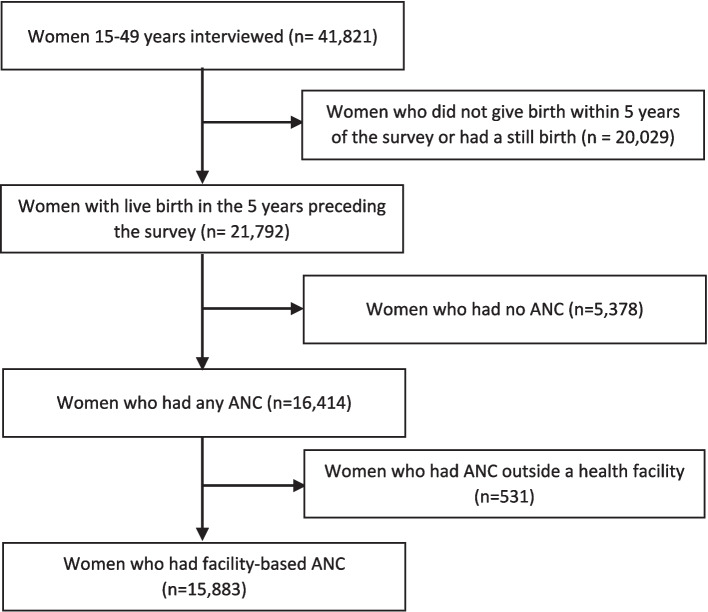
Flow diagram of inclusion into the analysis

Of the 41,821 women aged 15–49 years interviewed, our analysis included 21,792 women who had a live birth in the five years preceding the survey. Among them, 16,414 women reported having ANC during pregnancy for their most recent live birth, of which 15,883 women had facility-based ANC (Fig. 2). Among facility-based ANC users, 3,247 women had timely and adequate ANC visits.

### Characteristics of women with facility-based ANC

The sociodemographic characteristics of all women with live births are shown in Supplementary Table 1. Table 3 presents the sociodemographic characteristics of the 15,883 women who had at least one facility-based ANC and the 3,247 women who had timely and adequate ANC. Of the ANC users, 15.2% were from the poorest quintile, and 22.1% were from the richest. Most ANC users were in the 20–34-year age group during the pregnancy (74.9%), had wanted the baby when they became pregnant (87.5%), and received ANC services only in public facilities (81.0%). Over half of the ANC users (51.9%) were adolescents during their first pregnancy, and 22.7% had two to four previous births. At the time of the survey, most ANC users (94.0%) were in a union, and only 2.7% had health insurance. About half of the women (49.7%) attained secondary or higher levels of education, while 34.0% had no formal education. Most ANC users (53.2%) resided in rural areas, 30.3% were in the North-West region, and 55.8% identified as Muslims.

**Table 3:** Sociodemographic characteristics of women aged 15–49 years who had facility-based ANC

Characteristic	At least one ANC (*n* = 15,883)		Timely and adequate ANC (*n* = 3,247)	
	Unweighted frequency	Weighted %	Unweighted frequency	Weighted %
Household wealth quintile				
Poorest	2,579	15.2	311	8.4
Poor	3,206	19.2	512	13.9
Middle	3,610	21.6	702	19.6
Richer	3,458	21.9	769	22.7
Richest	3,030	22.1	953	35.4
ANC facility type				
Only public	13,033	81.0	2,318	68.2
Any private	2,850	19.0	929	31.8
Age group at childbirth				
15–19	1,466	9.2	272	7.9
20–34	11,814	74.9	2,441	76.1
35–49	2,603	15.9	534	16.0
Age at first pregnancy				
< 20	8,285	51.9	1,929	62.0
≥ 20	7,598	48.1	1,318	38.0
Marital status				
Currently in a union	14,857	94.0	3,034	93.6
Never/previously in a union	1,026	6.0	213	6.4
Parity				
0	2,928	18.6	754	24.6
1	2,896	19.1	685	22.7
2–4	6,385	39.6	1,282	38.2
5 or more	3,674	22.7	526	14.5
Desire for a baby				
Wanted then	13, 822	87.5	2,827	87.9
Wanted later	1,516	9.3	319	9.3
Wanted no more	545	3.2	101	2.8
Highest educational level				
None	5,333	34.0	639	19.0
Primary	2,704	16.3	509	14.0
Secondary	6,105	38.0	1,517	47.0
Higher	1,741	11.7	582	20.0
Health insurance				
Not covered	15,432	97.3	3,109	95.7
Covered	451	2.7	138	4.3
Place of residence				
Rural	9,254	53.2	1,699	44.5
Urban	6,629	46.8	1,548	55.5
Region				
North-Central	2,814	13.5	753	17.2
North-East	3,210	17.2	462	12.0
North-West	3,924	30.3	367	14.2
South-East	2,147	12.2	643	18.1
South-South	1,532	9.3	330	11.0
South-West	2,256	17.6	692	27.5
Religious affiliation				
Christianity	7,374	43.8	2,017	61.8
Islam	8,410	55.8	1,221	38.0
Traditional/others	99	0.5	9	0.2

^*^ANC Antenatal care

Notably, 35.4% of the timely and adequate ANC users were from the richest quintile, 68.2% had ANC in only public facilities, and 4.3% had health insurance. Over half of the women who had timely and adequate ANC (55.5%) resided in urban areas, 27.5% were in the South-West region, and 61.8% identified as Christians.

### Coverage of facility-based ANC and receipt of components related to maternal screening

Overall, 72.8% of women with live births in the recall period had ANC in health facilities. Of the ANC users, 14.7% had timely and adequate ANC visits (Table 4). All three maternal screening components were received at least once by 83.4% of ANC users: blood pressure was measured in 95.2%, blood sampling was done in 89.3%, and urine sampling was performed in 88.0%. Blood pressure measurement was the only screening component received by 4.3% of ANC users, while 2.9% did not receive any of the ANC components.

**Table 4:** Coverage of facility-based ANC and receipt of ANC components related to maternal screening

Outcome variable	Unweighted frequency	Weighted percentage, %
All women with a live birth (*n* = 21,792)		
Facility-based ANC	15,883	72.8
Women who had facility-based ANC (*n* = 15,883)		
Timely ANC	3,890	17.6
Adequate ANC	9,240	42.5
Timely and adequate ANC	3,247	14.7
Women who had facility-based ANC (*n* = 15,883)		
BP measured	15,142	95.2
Urine sample taken	13,888	88.0
Blood sample taken	14,139	89.3
*Combinations of ANC components:*		
None received	432	2.9
BP measured only	718	4.3
Urine sample taken only	71	0.4
Blood sample taken only	66	0.4
BP measured and urine sample taken	523	3.1
BP measured and blood sample taken	779	4.4
Urine and blood samples taken	172	1.1
All three components received	13,122	83.4
*Total*	15,883	100.0

^*^ANC Antenatal care, *BP* Blood pressure

**Fig. 3:**
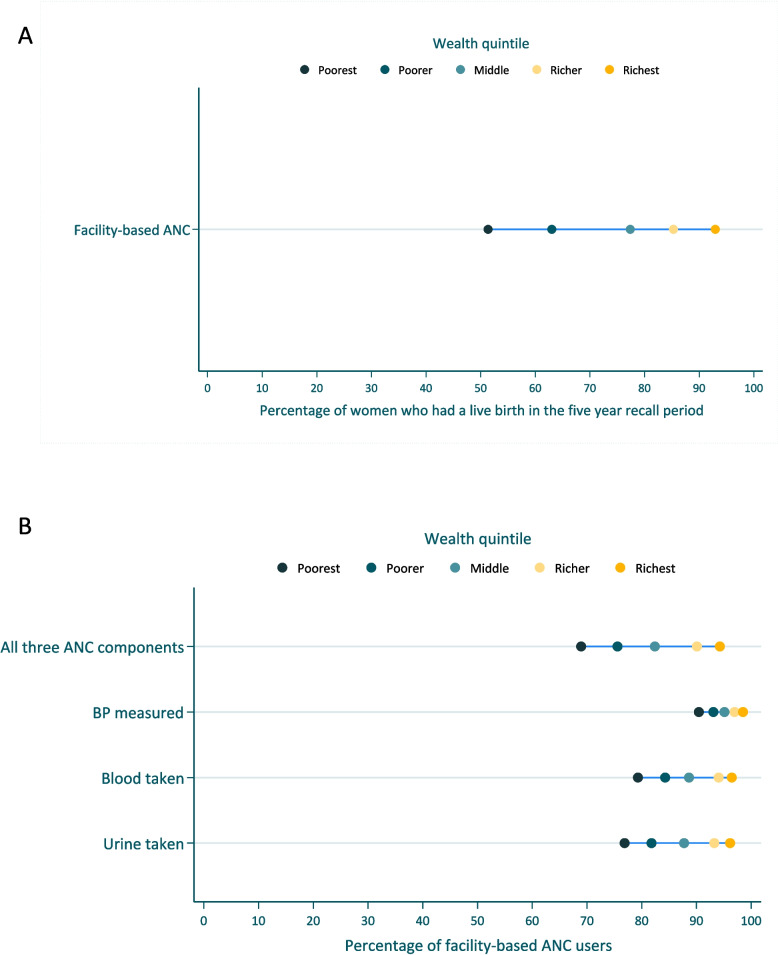
**A** Facility-based ANC coverage by wealth quintile in Nigeria (*n* = 21,792) **B.** Receipt of ANC components related to maternal screening by wealth quintile, among facility-based ANC users in Nigeria (*n* = 15,883); *BP – blood pressure*

Figure 3A shows the facility-based ANC coverage stratified by wealth quintile. More women from richer households (85.3%) had facility-based ANC than women from poorer households (63.0%). All three maternal screening components were received by 94.3% of ANC users from the richest quintile compared to 68.9% from the poorest quintile (Fig. 3B). Supplementary Fig. 1 presents the coverage data of maternal screening components stratified by wealth quintile for all women in need of ANC.

### Determinants of facility-based ANC use and receipt of maternal screening components

Table 5 presents the bivariate and multivariable logistic regression models examining the association between household wealth and facility-based ANC utilisation. In bivariate analysis, women from the richest quintile of households had significantly higher odds of receiving ANC in a health facility than from the poorest (OR = 12.48, 95% CI: 9.77–15.95). Women from the richest households maintained a significantly higher odds of having facility-based ANC compared to those from the poorest households after adjusting for age at most recent childbirth, history of adolescent pregnancy, marital status, parity, highest educational level, health insurance coverage, place of residence, region, and religious affiliation (aOR = 4.25, 95% CI: 3.23–5.59).

**Table 5:** Bivariate and multivariable logistic regression of factors associated with facility-based ANC use (*n* = 21,792) and receiving maternal screening components among facility-based ANC users (*n* = 15,883) in Nigeria

Characteristic	Facility-based ANC use		Receipt of three ANC screening components	
	Crude OR (95% CI)	Adjusted OR (95% CI)	Crude OR (95% CI)	Adjusted OR (95% CI)
Household wealth				
Poorest	Ref	Ref	Ref	Ref
Poorer	***1.61 (1.37–1.90)	***1.46 (1.24–1.71)	***1.39 (1.16–1.67)	*1.26 (1.05–1.50)
Middle	***3.24 (2.71–3.87)	***2.33 (1.93–2.81)	***2.11 (1.73–2.58)	***1.56 (1.26–1.92)
Richer	***5.49 (4.54–6.65)	***2.86 (2.32–3.54)	***4.10 (3.27–5.13)	***2.43 (1.90–3.11)
Richest	***12.48 (9.77–15.95)	***4.25 (3.23–5.59)	***7.43 (5.73–9.63)	***3.43 (2.52–4.68)
ANC facility type				
Only public	-	-	Ref	Ref
Any private	-	-	*1.20 (1.01–1.43)	***0.51 (0.43–0.61)
Timely and adequate ANC				
No	-	-	Ref	Ref
Yes	-	-	***1.83 (1.57–2.13)	***1.45 (1.24–1.68)
Age group at childbirth				
15–19	Ref	Ref	Ref	Ref
20–34	***1.68 (1.49–1.88)	***1.55 (1.32–1.83)	***1.65 (1.42–1.91)	1.18 (0.97–1.45)
35–49	***1.34 (1.17–1.54)	***1.52 (1.25–1.86)	***1.79 (1.49–2.14)	*1.40 (1.07–1.83)
Age at first pregnancy				
≥ 20	Ref	Ref	Ref	Ref
< 20	***0.46 (0.42–0.51)	1.07 (0.97–1.18)	***0.55 (0.49–0.62)	1.00 (0.87–1.14)
Marital status				
Not currently in union	Ref	Ref	Ref	Ref
Currently in union	0.89 (0.75–1.04)	**1.35 (1.12–1.63)	1.14 (0.94–1.40)	1.16 (0.95–1.40)
Parity				
0	Ref	Ref	Ref	Ref
1	0.91 (0.80–1.05)	***0.76 (0.64–0.89)	1.05 (0.90–1.22)	0.94 (0.80–1.11)
2–4	***0.80 (0.71–0.89)	***0.72 (0.61–0.85)	1.03 (0.90–1.17)	1.02 (0.87–1.19)
5 or more	***0.50 (0.44–0.56)	***0.66 (0.56–0.79)	***0.74 (0.64–0.86)	0.95 (0.77–1.18)
Educational level				
None	Ref	Ref	Ref	Ref
Primary	***2.99 (2.59–3.46)	***2.57 (2.22–2.98)	***1.44 (1.22–1.69)	1.16 (0.99–1.37)
Secondary	***5.29 (4.58–6.11)	***3.59 (3.10–4.16)	***2.49 (2.14–2.89)	***1.62 (1.37–1.92)
Higher	***28.53 (20.33–40.06)	***12.71 (8.83–18.28)	***6.90 (5.00–9.51)	***2.85 (2.04–4.00)
Health insurance				
Not covered	Ref	Ref	Ref	Ref
Covered	**3.35 (1.52–7.41)	1.20 (0.67–2.15)	***3.15 (1.92–5.19)	1.24 (0.73–2.09)
Place of residence				
Rural	Ref	Ref	Ref	Ref
Urban	***3.32 (2.80–3.92)	*1.25 (1.04–1.51)	***3.02 (2.51–3.62)	***1.62 (1.31–2.01)
Region				
North-Central	Ref	Ref	Ref	Ref
North-East	1.01 (0.79–1.28)	***2.24 (1.76—2.85)	***0.30 (0.23–0.38)	***0.35 (0.27—0.45)
North-West	**0.70 (0.56–0.87)	***1.54 (1.23—1.93)	***0.51 (0.40–0.66)	***0.56 (0.42—0.75)
South-East	***3.99 (2.95–5.41)	*1.38 (1.01—1.88)	0.78 (0.58–1.04)	***0.57 (0.42—0.77)
South-South	1.16 (0.91–1.47)	***0.38 (0.29—0.49)	***0.59 (0.45–0.78)	***0.39 (0.29—0.51)
South-West	***2.77 (2.09–3.66)	0.98 (0.74—1.30)	**1.46 (1.10–1.94)	0.75 (0.56—1.02)
Religious affiliation				
Christianity	Ref	Ref	Ref	Ref
Islam	***0.38 (0.33–0.44)	***0.70 (0.57—0.86)	***0.73 (0.62–0.86)	**1.34 (1.10—1.62)
Traditional/others	***0.33 (0.21–0.51)	0.74 (0.40—1.37)	0.85 (0.48–1.48)	1.58 (0.75—3.30)

*ANC *Antenatal care, *OR *Odds ratio, *CI *Confidence interval

***p < 0.001, **p < 0.01*, *and *p < 0.05

Among facility-based ANC users, women from the richest quintile had significantly higher odds of receiving all three screening components than those from the poorest quintile (OR = 7.43, 95% CI: 5.73–9.63). This association remained significant after adjusting for facility type and other confounders (aOR = 3.43, 95% CI: 2.52–4.68). In bivariate analysis, women who had ANC in any private facility had slightly higher odds of receiving all screening components compared to those in only public facilities (OR = 1.20, 95% CI: 1.01–1.43). However, multivariable analysis revealed that women who had ANC in any private facility had 49% lower adjusted odds of receiving all screening components compared to women with ANC in only public facilities after adjusting for confounders (aOR = 0.51, 95% CI: 0.43–0.61). There was no evidence of interaction between household wealth and facility type on the receipt of all screening components in the adjusted model (Wald test *p* = 0.413). All variables were maintained in the multivariable models, with no significant evidence of multicollinearity (VIF less than 2 for all variables).

Supplementary Table 2 presents the results of bivariate and multivariable analyses examining the association between household wealth and each component of ANC screening. Women from the richest quintile of households also had significantly higher adjusted odds of receiving individual ANC screening components compared to those from the poorest quintile [blood pressure measured (aOR = 3.14, 95% CI: 1.95–5.06); urine sample taken (aOR = 4.27, 95% CI: 3.08–5.92); and blood sample taken (aOR = 3.00, 95% CI: 2.06–4.36)]. There were significantly lower odds of receiving the individual components related to maternal screening during ANC in any private facility than in only public facilities [blood pressure measured (aOR = 0.47, 95% CI: 0.36–0.61); urine sample taken (aOR = 0.50, 95% CI: 0.41–0.61); and blood sample taken (aOR = 0.47, 95% CI: 0.38–0.58)].

## Discussion

This study analysed the 2018 NDHS data to estimate coverage and examine the determinants of facility-based ANC use and receipt of ANC components related to maternal screening for common pregnancy conditions in Nigeria. We found that seven in 10 women had ANC in a health facility, and eight in 10 ANC users received all three maternal screening components. Blood pressure measurement was the most frequently reported screening component, followed by blood and urine collection. Women from the richest households had four times the odds of attending ANC in a health facility, and among ANC users, three times the odds of receiving all ANC components compared to the poorest women. The odds of receiving all ANC components were 49% lower for women who had ANC visits in any private health facility compared to those who visited only public health facilities.

Our study estimated the facility-based ANC coverage in Nigeria at 72.8%. This coverage is slightly higher than the 67% ANC coverage reported in the 2018 NDHS due to a difference in the definition of ANC use [11]. While the NDHS report defined ANC use as care received from a skilled healthcare professional, we reported facility-based ANC because the ANC components related to maternal screening require the use of equipment and materials that are mainly available in health facilities. Another reason for choosing this definition was that the survey participants were more likely to accurately recall the place of ANC than accurately identify the health professional(s) seen during ANC. ANC coverage in Nigeria remains among the lowest in sub-Saharan African countries, as a recent study indicates a range from 62.9% to 99.3% [25]. This finding highlights significant gaps in achieving the SDG target of universal health coverage [26]. Furthermore, the coverage of the WHO-recommended early initiation of ANC in the first trimester and a minimum of four ANC visits was even lower. We found that only about 15% of women had timely and adequate ANC. This also ranks among the lowest reported across low- and middle-income countries (LMICs), which ranged from 14.9% to 89.1% [27]. Early initiation of ANC and an adequate number of visits are important to ensure the timely detection of pregnancy-related conditions through screening interventions, thereby preventing adverse maternal outcomes.

Regarding the maternal screening components of care received during ANC, we found that 83% of ANC users received all three ANC components, with blood pressure measurement the most reported (95.2%) and urine sample collection the least performed (88.0%). These findings are consistent with the NDHS 2018 report despite the difference in the ANC definition [11]. Our findings also align with a study that reported > 80% blood pressure measurement in women with timely and adequate ANC in Nigeria and other LMICs [27]. Urine sample collection was the least received ANC screening component, despite also being an intervention expected to be performed at every ANC visit, similar to blood pressure measurement. A study on DHS data from Burundi similarly found that urine sample collection was the least reported ANC component [28]. The lack of required consumables and inadequate human resources to offer urine testing regularly could explain its relatively lower coverage compared to other screening interventions. A similar plausible explanation could be provided for the coverage of blood sample collection. However, the wider range of specific screening interventions performed on blood samples may likely account for its higher coverage than urine sample collection. Also, the presence of vertical programs offering free voluntary HIV screening during ANC could explain the relatively high coverage of blood sample collection.

We evaluated the association of household wealth with the utilisation of facility-based ANC and the receipt of ANC components related to screening. Our findings revealed that women from the richest quintile were more likely to have facility-based ANC. This confirms findings from previous studies in Nigeria, which identified household wealth as a predictor of ANC use [12, 29, 30]. Furthermore, we found that household wealth also influences the receipt of maternal screening components during ANC: women from the richest quintile were up to three times more likely to receive all maternal screening components than those from the poorest. Household wealth is directly linked to the affordability and accessibility of quality healthcare services [16, 29]. More women from the richest wealth quintile had timely and adequate ANC visits than women from the poorest quintile, which could explain the receipt of more screening components [31]. Early initiation of ANC increases the possibility of having more ANC visits, thereby increasing the opportunity to receive more ANC components [32]. However, even after controlling for timely and adequate ANC visits, the wealth disparity in receiving ANC screening components persisted, highlighting the independent effect of household wealth. Together, these findings imply that wealth inequality is associated with a ‘double penalty’ on the poorest women, who often have more need for but are the least likely to use ANC and to receive its components [33]. Also, women from the poorest households are still less likely to get all three screening components, even in public health facilities expected to provide equitable care. These disparities in maternal health service utilisation contribute to the high maternal morbidity and mortality in Nigeria. Addressing these socioeconomic disparities by focusing interventions on the economically disadvantaged population will help prevent avoidable maternal deaths.

Furthermore, we investigated the association between facility type and receipt of ANC components, which revealed that women were significantly less likely to receive the screening components if, during ANC, they used any private health facility. In the Nigerian health system, about 70% of maternal health services are provided by public health facilities [34]. The out-of-pocket payment in private health facilities partly explains the lower ANC components received in these facilities [22, 23]. The quality of care provided between public and private health facilities in LMICs is comparable [35]. While the experience of care between both facility types is largely similar, the provision of care is described as mixed. Elucidating the barriers to receiving all ANC screening components in the health facilities warrants further investigation. Qualitative studies to further understand the determinants of maternal screening during ANC in health facilities are necessary to help identify the factors limiting the implementation of routine screening services in the Nigerian health system.

### Strengths and limitations

A strength of this analysis is the large, nationally representative sample, which has a high response rate (99%) and is also sufficient to provide regional and statewide estimates, making the findings generalisable. The presence of sociodemographic and socio-economic characteristics in the dataset allowed us to control for multiple factors in investigating the main exposures of interest, thereby reducing bias from confounding. However, other sources of bias in this study design, such as residual confounding, measurement error, and selection bias, may potentially remain. Despite these strengths, our study had some limitations. We used the available data on ANC components received as proxies for maternal screening. Although this offers useful information on screening components of ANC, apart from blood pressure measurement and perhaps urine collection, they do not assess screening for specific pregnancy-related conditions. Also, women were asked if they had received the components at least once. Some of the screening components are recommended to be performed at each visit, while others have specific time intervals. This study could not establish whether sufficient screening interventions were performed. We also cannot determine if the women received quality and adequate maternal screening or if the necessary interventions were carried out after detecting pregnancy-related conditions. The validity of the women’s recall of receiving the various screening components is also subject to variability, with more invasive procedures, such as taking a blood sample, being more easily remembered than, for example, checking blood pressure or providing a urine sample [36]. Other routine screening interventions, such as weight and height measurements, were not captured in the 2018 NDHS. However, this analysis offers valuable insights into the screening components of care provided in the health facilities. Finally, the most recent NDHS was conducted in 2023–24, with the resulting data due for release in 2025. However, the funding cuts announced by the US government halted the program in early 2025, preventing the release and public use of the data [37]. Nonetheless, findings obtained from the 2018 NDHS are useful in understanding the factors associated with maternal screening during ANC and developing interventions to address maternal health disparities. With the recent official release of the 2024 NDHS final report and accompanying dataset, it will be good to see if the pattern has changed [38].

## Conclusion

This study assessed the coverage and determinants of receipt of maternal screening during ANC in Nigeria with a focus on wealth inequalities and facility type. The findings indicate inequities in facility-based ANC and receipt of maternal screening based on wealth inequalities. Public facilities provided maternal screening services more readily than private health facilities, but not more equitably. Socioeconomic inequalities can be mitigated by the health system through the provision of appropriate care components regardless of social class or status, or facility type. Concerted efforts are required to regulate the private facilities and strengthen the public facilities to provide high-quality maternal healthcare services. There is a need to close the gap in access to maternal health services in Nigeria by targeting the poorest women who need ANC the most, if we are to achieve universal health coverage— “leave no one behind”— and avoid preventable maternal deaths towards SDG targets.

## Supplementary Information

Supplementary Material 1.

## Data Availability

The data analysed in this study were obtained from Measure DHS and are not permitted to be shared in a public repository.

## Abbreviations

ANC: Antenatal care
BP: Blood pressure
CI: Confidence Interval
HIV: Human Immunodeficiency Virus
LMICs: Low- and middle-income countries
LSHTM: London School of Hygiene and Tropical Medicine
MMR: Maternal Mortality Ratio
NDHS: Nigeria Demographic and Health Survey
OR: Odds ratio
SDG: Sustainable Development Goal
US: United States
VIF: Variance Inflation Factor
WHO: World Health Organization
