# Supplementary material for: Maternal screening coverage and determinants during facility-based antenatal care in Nigeria: analysis of the 2018 Demographic and Health Survey
Source: Reprod Health. 2026 Jan 28;23:49. doi: 10.1186/s12978-026-02269-1 (PMC12924446; doi:10.1186/s12978-026-02269-1)
Supplement: Supplementary file 1 — Supplementary Material 1. [file 12978_2026_2269_MOESM1_ESM.docx]

Supplementary File

**Supplementary Table 1**. Sociodemographic characteristics of all women aged 15-49 years who had a live birth within 5 years preceding the survey (n = 21,792)

| Characteristic | In need of ANC (n = 21,792) | | Timely and adequate ANC (n = 3,247) | |
| --- | --- | --- | --- | --- |
|  | Unweighted frequency | Weighted % | Unweighted frequency | Weighted % |
| **Household wealth quintile** |  |  |  |  |
| Poorest | 5,025 | 21.5 | 311 | 8.4 |
| Poor | 4,905 | 22.1 | 512 | 13.9 |
| Middle | 4,586 | 20.3 | 702 | 19.6 |
| Richer | 4,025 | 18.7 | 769 | 22.7 |
| Richest | 3,251 | 17.3 | 953 | 35.4 |
| **ANC facility type** |  |  |  |  |
| Only public | - | - | 2,318 | 68.2 |
| Any private | - | - | 929 | 31.8 |
| **Age group at childbirth** |  |  |  |  |
| 15-19 | 2,264 | 10.4 | 272 | 7.9 |
| 20-34 | 15,860 | 73.0 | 2,441 | 76.1 |
| 35-49 | 3,668 | 16.6 | 534 | 16.0 |
| **Age at first pregnancy** |  |  |  |  |
| <20 | 12,392 | 56.8 | 1,929 | 62.0 |
| ≥20 | 9,400 | 43.2 | 1,318 | 38.0 |
| **Marital status** |  |  |  |  |
| Currently in a union | 20,419 | 94.2 | 3,034 | 93.6 |
| Never/previously in a union | 1,373 | 5.8 | 213 | 6.4 |
| **Parity** |  |  |  |  |
| 0 | 3,753 | 17.3 | 754 | 24.6 |
| 1 | 3,811 | 18.0 | 685 | 22.7 |
| 2-4 | 8,577 | 38.9 | 1,282 | 38.2 |
| 5 or more | 5,651 | 25.8 | 526 | 14.5 |
| **Desire for a baby** |  |  |  |  |
| Wanted then | 19, 054 | 87.9 | 2,827 | 87.9 |
| Wanted later | 1,976 | 8.8 | 319 | 9.3 |
| Wanted no more | 762 | 3.3 | 101 | 2.8 |
| **Highest educational level** |  |  |  |  |
| None | 9,527 | 44.4 | 639 | 19.0 |
| Primary | 3,410 | 15.0 | 509 | 14.0 |
| Secondary | 7,064 | 31.8 | 1,517 | 47.0 |
| Higher | 1,791 | 8.8 | 582 | 20.0 |
| **Health insurance status** |  |  |  |  |
| Not covered | 21,298 | 97.8 | 3,109 | 95.7 |
| Covered | 494 | 2.2 | 138 | 4.3 |
| **Place of residence** |  |  |  |  |
| Rural | 14,082 | 60.2 | 1,699 | 44.5 |
| Urban | 7,710 | 39.8 | 1,548 | 55.5 |
| **Region** |  |  |  |  |
| North Central | 3,875 | 13.8 | 753 | 17.2 |
| North East | 4,506 | 17.6 | 462 | 12.0 |
| North West | 6,309 | 34.9 | 367 | 14.2 |
| South East | 2,365 | 9.8 | 643 | 18.1 |
| South South | 2,174 | 9.2 | 330 | 11.0 |
| South West | 2,563 | 14.7 | 692 | 27.5 |
| **Religious affiliation** |  |  |  |  |
| Christianity | 8,929 | 38.1 | 2,017 | 61.8 |
| Islam | 12,687 | 61.4 | 1,221 | 38.0 |
| Traditional/others | 176 | 0.5 | 9 | 0.2 |

**Supplementary Figure 1.** Receipt of ANC component related to maternal screening by wealth quintile amongst all women in need of ANC in Nigeria (NDHS 2018)

**Supplementary Table 2**. Bivariate and multivariable logistic regression of factors associated with receiving each content of ANC related to maternal screening in Nigeria (NDHS 2018)

| Characteristic | Blood pressure measured | | Urine sample taken | | Blood sample taken | |
| --- | --- | --- | --- | --- | --- | --- |
|  | Crude OR (95% CI) | Adjusted OR (95% CI) | Crude OR (95% CI) | Adjusted OR (95% CI) | Crude OR (95% CI) | Adjusted OR (95% CI) |
|  |  |  |  |  |  |  |
| **Household wealth** |  |  |  |  |  |  |
| Poorest | Ref | Ref | Ref | Ref | Ref | Ref |
| Poorer | 1.43 [1.06 – 1.91] | 1.37 [1.03 – 1.82] | 1.35 [1.11 – 1.65] | 1.23 [1.01 – 1.49] | 1.40 [1.14 – 1.72] | 1.26 [1.02– 1.55] |
| Middle | 2.06 [1.51 – 2.80] | 1.64 [1.19 – 2.27] | 2.15 [1.72 – 2.70] | 1.73 [1.37 – 2.18] | 2.04 [1.61 – 2.57] | 1.46 [1.14 – 1.87] |
| Richer | 3.37 [2.39 – 4.74] | 2.07 [1.46 – 2.93] | 4.16 [3.22 – 5.38] | 2.90 [2.24 – 3.76] | 4.13 [3.14 – 5.44] | 2.30 [1.68 – 3.14] |
| Richest | 6.93 [4.55 – 10.54] | 3.14 [1.95 – 5.06] | 7.52 [5.71 – 9.90] | 4.27 [3.08 – 5.92] | 7.08 [5.23 – 9.60] | 3.00 [2.06 – 4.36] |
| **ANC facility type** |  |  |  |  |  |  |
| Only public | Ref | Ref | Ref | Ref | Ref | Ref |
| Any private | 1.12 [0.87 – 1.45] | 0.47 [0.36 – 0.61] | 1.08 [0.89 – 1.31] | 0.50 [0.41 – 0.61] | 1.18 [0.97 – 1.43] | 0.47 [0.38 – 0.58] |
| **Timely and adequate ANC** |  |  |  |  |  |  |
| No | Ref | Ref | Ref | Ref | Ref | Ref |
| Yes | 1.88 [1.45 – 2.43] | 1.39 [1.07 – 1.81] | 1.93 [1.62 – 2.30] | 1.54 [1.29 – 1.83] | 1.76 [1.48 – 2.09] | 1.37 [1.15 – 1.65] |
| **Age at interview** |  |  |  |  |  |  |
| 15-19 | Ref | Ref | Ref | Ref | Ref | Ref |
| 20-29 | 1.63 [1.27 – 2.09] | 1.37 [1.00 – 1.88] | 1.43 [1.19 – 1.71] | 1.15 [0.92 – 1.45] | 1.60 [1.33 – 1.92] | 1.12 [0.89 – 1.42] |
| 30-39 | 2.12 [1.65 – 2.73] | 1.81 [1.22 – 2.69] | 1.58 [1.31 – 1.90] | 1.31 [0.99 – 1.73] | 1.94 [1.60 – 2.36] | 1.17 [0.88 – 1.56] |
| 40-49 | 1.76 [1.17 – 2.65] | 1.90 [1.07 – 3.36] | 1.31 [0.99 – 1.72] | 1.34 [0.92 – 1.96] | 1.49 [1.14 – 1.95] | 1.04 [0.70 – 1.54] |
| **Age at first pregnancy** |  |  |  |  |  |  |
| No teenage | Ref | Ref | Ref | Ref | Ref | Ref |
| Teenage | 0.54 [0.45 – 0.64] | 1.09 [0.88 – 1.34] | 0.59 [0.52 – 0.67] | 1.05 [0.90 – 1.21] | 0.55 [0.48 – 0.62] | 0.98 [0.84 – 1.14] |
| **Marital status** |  |  |  |  |  |  |
| Not currently in union | Ref | Ref | Ref | Ref | Ref | Ref |
| Currently in union | 1.25 [0.90 – 1.72] | 1.44 [1.04 – 2.00] | 1.15 [0.92– 1.45] | 1.12 [0.89 – 1.41] | 1.20 [0.93– 1.53] | 1.25 [0.98 – 1.59] |
| **Parity** |  |  |  |  |  |  |
| 0 | Ref | Ref | Ref | Ref | Ref | Ref |
| 1 | 1.09 [0.83 – 1.43] | 0.90 [0.67 – 1.23] | 1.11 [0.93– 1.32] | 1.00 [0.82 – 1.21] | 1.06 [0.89 – 1.28] | 0.96 [0.79 – 1.16] |
| 2-4 | 1.03 [0.81 – 1.31] | 0.87 [0.63 – 1.20] | 0.95 [0.82 – 1.09] | 0.92 [0.77 – 1.11] | 1.05 [0.90 – 1.23] | 1.05 [0.87 – 1.27] |
| 5 or more | 0.79 [0.62 – 1.02] | 0.78 [0.51 – 1.20] | 0.69 [0.58 – 0.81] | 0.84 [0.65 – 1.08] | 0.83 [0.70 – 0.99] | 1.16 [0.88 – 1.52] |
| **Educational level** |  |  |  |  |  |  |
| None | Ref | Ref | Ref | Ref | Ref | Ref |
| Primary | 1.30 [0.99 – 1.70] | 1.01 [0.76 – 1.34] | 1.35 [1.12 – 1.62] | 1.18 [0.98 – 1.41] | 1.57 [1.30 – 1.90] | 1.27 [1.05 – 1.54] |
| Secondary | 2.58 [2.03 – 3.27] | 1.61 [1.21 – 2.13] | 2.42 [2.04 – 2.87] | 1.68 [1.38 – 2.06] | 2.57 [2.16 – 3.06] | 1.69 [1.38 – 2.05] |
| Higher | 7.27 [3.58 – 14.76] | 2.91 [1.33 – 6.37] | 7.40 [5.20 – 10.51] | 3.16 [2.17 – 4.60] | 7.59 [4.95– 11.65] | 3.33 [2.12 – 5.23] |
| **Health insurance** |  |  |  |  |  |  |
| Not covered | Ref | Ref | Ref | Ref | Ref | Ref |
| Covered | 1.85 [0.79 – 4.35] | 0.75 [0.30 – 1.83] | 2.56 [1.47 – 4.48] | 0.91 [0.50 – 1.65] | 3.33 [1.64 – 6.74] | 1.34 [0.65 – 2.75] |
| **Place of residence** |  |  |  |  |  |  |
| Rural | Ref | Ref | Ref | Ref | Ref | Ref |
| Urban | 3.01 [2.29 – 3.95] | 1.75 [1.29 – 2.39] | 2.87 [2.33 – 3.52] | 1.64 [1.30 – 2.07] | 2.98 [2.39 – 3.71] | 1.51 [1.15 – 1.98] |
| **Region** |  |  |  |  |  |  |
| North-Central | Ref | Ref | Ref | Ref | Ref | Ref |
| North-East | 0.71 [0.49 – 1.04] | 1.05 [0.71 – 1.54] | 0.26 [0.20 – 0.36] | 0.34 [0.26 – 0.46] | 0.35 [0.27 – 0.46] | 0.40 [0.30 – 0.53] |
| North-West | 0.57 [0.41 – 0.79] | 0.80 [0.55 – 1.17] | 0.44 [0.33 – 0.59] | 0.55 [0.40 – 0.76] | 0.61 [0.46 – 0.81] | 0.64 [0.46 – 0.88] |
| South-East | 1.20 [0.83 – 1.75] | 0.71 [0.47 – 1.07] | 0.51 [0.37 – 0.70] | 0.32 [0.23 – 0.44] | 1.22 [0.90 – 1.67] | 1.00 [0.72 – 1.40] |
| South-South | 0.73 [0.50 – 1.07] | 0.39 [0.26 – 0.59] | 0.45 [0.33 – 0.61] | 0.25 [0.18 – 0.34] | 0.80 [0.59 – 1.09] | 0.56 [0.40 – 0.77] |
| South-West | 2.29 [1.42 – 3.69] | 1.19 [0.74 – 1.91] | 0.98 [0.72 – 1.33] | 0.45 [0.33 – 0.61] | 1.77 [1.27 – 2.46] | 1.01[0.72 – 1.42] |
| **Religious affiliation** |  |  |  |  |  |  |
| Christianity | Ref | Ref | Ref | Ref | Ref | Ref |
| Islam | 0.57 [0.45 – 0.71] | 0.71 [0.52 – 0.97] | 0.76 [0.64 – 0.92] | 1.13 [0.92 – 1.40] | 0.69 [0.57 – 0.83] | 1.39 [1.10 – 1.74] |
| Traditional/others | 0.40 [0.16 – 0.99] | 0.69 [0.24 – 1.97] | 0.81 [0.44 – 1.52] | 1.45 [0.75 – 2.82] | 1.49 [0.51 – 4.36] | 2.78 [0.79 – 9.71] |

*ANC – Antenatal care, OR – Odds ratio, CI – Confidence interval. ***p<0.001, **p<0.01, and *p<0.05*
